# Urinary tract infection among primigravid singleton pregnancies: a retrospective study from the North of Jordan

**DOI:** 10.3389/fgwh.2025.1654691

**Published:** 2025-09-18

**Authors:** Reem Hatamleh, Nemeh Al-Akour, Wafa'a Al-Bakheet, Maha Atout, Majeda El-Banna

**Affiliations:** 1 Department of Maternal and Child Health, Faculty of Nursing, Jordan University of Science and Technology, Irbid, Jordan; 2 Faculty of Nursing, Philadelphia University, Amman, Jordan; 3 College of Nursing, QU Health, Qatar University, Doha, Qatar

**Keywords:** primigravida, singleton pregnancy, urinary tract infection, pregnancy, Jordan, maternal health, risk factors

## Abstract

**Objectives:**

Urinary tract infections (UTIs) are common during pregnancy and can lead to adverse maternal and neonatal outcomes. Despite their significance, data on UTI prevalence and associated factors among pregnant women in Jordan remain limited. This study aimed to determine the prevalence of UTI and its associated factors among pregnant women in the northern Jordan.

**Methods:**

We conducted a retrospective study using a convenience sampling method to review the records of pregnant women registered in the King Abdullah University Hospital database between January 2017 and January 2020.

**Results:**

The study found that the prevalence of urinary tract infection (UTI) was 8.9%. Positive predictors included an interaction between chronological age and level of education (OR = 1.139; 95% CI: 1.040–1.248; *p* = 0.01), being in the third trimester of pregnancy (OR = 1.856; 95% CI: 1.052–3.273; *p* = 0.03), prolonged hospitalization (OR = 6.784; 95% CI: 4.075–11.292; *p* < 0.001), and anemia (OR = 3.662; 95% CI: 2.185–6.138; *p* < 0.001). Negative predictors included having a university degree (OR = 0.027; 95% CI: 0.002–0.344; *p* = 0.01) and being at the younger or older age during pregnancy (OR = 0.904; 95% CI: 0.839–0.974; *p* = 0.01).

**Conclusion:**

The study concluded that the prevalence of UTI among singleton primigravida pregnant women was high, given the potential consequences of infection during pregnancy. Therefore, appropriate interventional measures should be implemented by the government and relevant stakeholders to reduce the prevalence of the infection and its associated complications.

## Introduction

The American Urological Association (AUA) describes a urinary tract infection as the presence of pathogens in the urinary system, along with symptoms or signs of inflammation that require treatment (1).

UTIs are common during pregnancy, affecting nearly one in four women worldwide (23.9%) according to recent estimates (2). In pregnant women, asymptomatic bacteriuria is found in about 2%–10%, while more severe infections such as cystitis or pyelonephritis occur in 1%–2%.

Screening for ASB early in pregnancy can reduce the risk of serious complications, including preterm birth (3). After anemia, UTIs are the second most frequent cause of health problems for mothers and newborns (4, 5). If untreated, they can contribute to both maternal and neonatal illness and even death.

Asymptomatic bacteriuria (ASB) is usually diagnosed when two consecutive clean-catch urine samples each show at least 100,000 colony-forming units (CFU), or when a single midstream sample shows more than 100,000 bacteria per milliliter (6). Globally, ASB in pregnancy affects about 2%–15% of women, underscoring the need for timely testing and treatment.

Recent research indicates that urinary tract infections remain common throughout pregnancy across many contexts. A 2023 meta-analysis indicated a global prevalence of 23.9% (2). In Latin America, 7.54% of pregnant women experienced lower urinary tract infections, while 18.45% exhibited asymptomatic bacteriuria (7). A 2024 study in Saudi Arabia found that 4% of individuals were asymptomatic, with a prevalence of 5% (95% CI: 3.6–6.4) (8). These findings indicate that pregnancy-related UTIs remain a persistent and, in certain regions, growing problem.

Pregnancy presents considerable hazards for both the mother and fetus, with numerous risk factors increasing the probability of a urinary tract infection (UTI). The factors include low socioeconomic status, particular sexual behaviors, advanced maternal age, multiparity, structural urinary tract anomalies, sickle cell disease, diabetes, a previous history of urinary tract infections, lifestyle influences such as diet and clothing during pregnancy, and anemia (9–11). A UTI during pregnancy can pose considerable health risks for both the mother and the newborn (12).

In mothers, urinary tract infections can result in premature rupture of membranes, anemia, miscarriage, hypertension, and pyelonephritis. The fetus is at risk for low birth weight and premature birth (13–15). These complications not only have direct health impacts but also impose additional strain on healthcare systems, families, and communities (16).

Pregnancy-related disorders can adversely affect the health of both the mother and the fetus, such as urinary tract infections, which are associated with significant morbidity. In 2018, Jordan's maternal mortality rate was 29.6 per 100,000 live births, with hemorrhage being the predominant cause of maternal death, and sepsis accounting for 7.1% of maternal deaths (17).

This study looks at how common UTIs are in pregnancy and which social or obstetric factors are linked to them. Our aim is to give healthcare providers and decision-makers in Jordan practical information they can use to improve screening and management, with the ultimate goal of reducing infection rates and improving outcomes for mothers and infants. A thorough screening of urinary tract infections in pregnancy can elevate healthcare service quality, improve the health behaviors of pregnant women, and potentially reduce maternal and newborn morbidity and mortality associated with urinary tract infections. Furthermore, the study's results may enhance childbirth outcomes for Jordanian women and alleviate the strain of UTIs on healthcare systems.

Urinary tract infections in pregnancy are known to have adverse effects; however, recent region-specific data in Jordan are few, particularly concerning primigravida women with singleton pregnancies, a group that may possess distinct risk profiles and medical needs. Previous Jordanian research is less relevant to this group due to their frequent inclusion of mixed parity groups or inadequate analysis of sociodemographic and obstetric variables. Formulating targeted screening and prevention measures necessitates addressing this gap. This study aimed to determine the prevalence of UTI and its associated factors among pregnant women in the northern Jordan.

## Methods

### Research design

This study employed a retrospective research design, reviewing medical records to evaluate the prevalence of urinary tract infections (UTIs) in primigravida females with singleton fetuses and to examine the correlation between UTIs and various sociodemographic and obstetrical factors.

### Study setting

Jordan is a Middle Eastern country located in southwest Asia whose estimated population in 2023 was 11,439,213 6.5 million Jordanians (18). The study was conducted in King Abdullah University Hospital in Irbid (Jordan) which is the largest teaching hospital in the northern region of Jordan and a comprehensive maternity referral centre. This hospital was selected as the study setting for the current research because its maternity wards serve a substantial number of pregnant women (approximately 3200 births annually). Furthermore, the hospital employs fifty midwives and thirty registered nurses in its maternity wards and provides a broad range of pre-and post-natal services (such as labour wards and clinics).

### Participants

#### Inclusion and exclusion criteria

The eligibility criteria for this study are as follows: participants must be primigravida women with a singleton foetus who attended a prenatal clinic and delivered within the study period. Only women with free medical and obstetric histories were included. However, pregnancy-related complications, including anaemia, were documented when they occurred. The exclusion criteria encompass multigravida women, women with high-risk pregnancies or medical issues, and women who delivered in hospitals, and women with asymptomatic bacteriuria (≥100,000 CFU/ml without urinary tract infection symptoms), in order to focus exclusively on symptomatic UTIs. These criteria were chosen to minimize confounding variables associated with pre-existing maternal medical problems or prior obstetric history.

#### Sampling

All eligible pregnant women with singleton pregnancies who met the inclusion criteria were invited to participate in the study in the order that they were admitted between January 2017 and January 2020. This method is known as consecutive sampling, or total enumeration sampling. By including all available participants, this non-probability sampling technique, which is frequently employed in retrospective-based record studies, has the benefit of reducing bias (19). The medical records were reviewed and data were gathered from August 2019 until April 2020.

**Figure 1:**
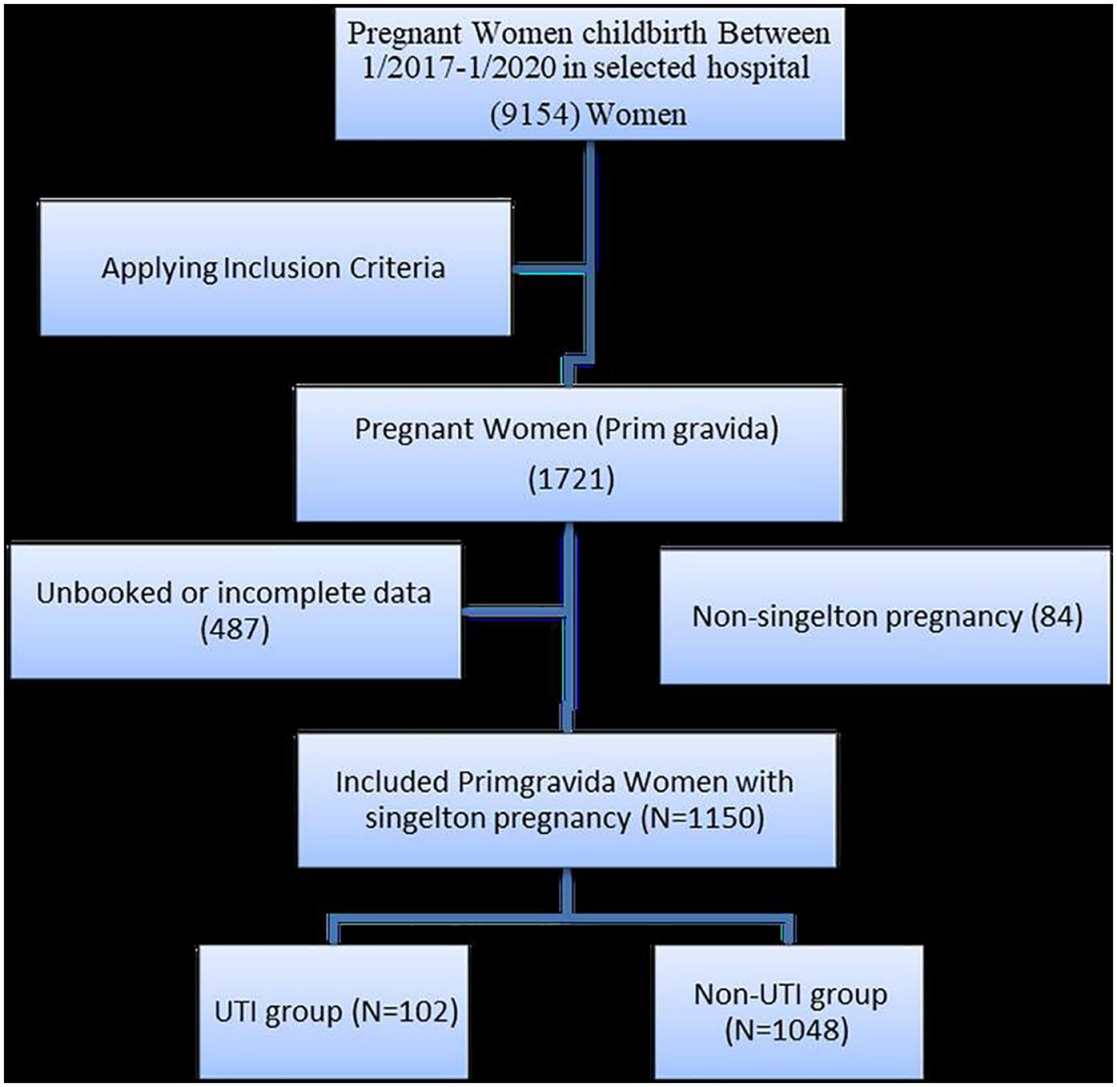
PRISMA flow diagram of the study sample selection process.

As illustrated by Figure 1, during the study period, 1,721 primigravida women were admitted to the maternity units. Of these, 487 were excluded due to a lack of data concerning their health status during pregnancy and an additional 84 participants with non-singleton pregnancies (i.e., twins, triplets, and quadruplets) were excluded from the study. This process was conducted between January 2017 and January 2020 and resulted in a total study population of 1,150 primigravida women (102 women with urinary tract infections and 1,048 women without urinary tract infections).

#### Sample description

The total number of primigravida women during the study period was 1,721. Four hundred and eighty-seven had incomplete or no data, and 84 had multiple foetuses/pregnancies and were excluded; therefore, the total sample of primigravida who met the inclusion criteria was 1,150, of which 102 had UTIs, while the rest did not (*n* = 1,048).

### Ethical considerations

Approval to conduct the study was obtained from the Institutional Review Board at Jordan University of Science and Technology (approval number JUST 638/2019). All of the maternal and newborn medical records were obtained with the assistance of the hospital's medical records department. The researcher conducted a comprehensive review of each participant's paper-based and computer-based records and extracted the required data via an anonymously coded data abstraction form. To ensure patient confidentiality and adhere to data protection requirements, all of the medical files were reviewed in a designated private space in the medical records department. All of the retrieved coded data was entered into specified files for processing by SPSS software and the collated data was securely stored on the researcher's password-protected personal computer to prevent access by anyone other than the research team.

### Data collection procedure

Following approval from the Institutional Review Board at Jordan University of Science and Technology, the data collection phase of the study commenced. Delivery books were used to identify all primigravida and singleton pregnancies that occurred during the pre-determined study period, and their medical records were examined to establish whether each participant met the study's eligibility criteria. In the next phase of the data collection process, the candidates were divided into two groups (urinary tract infection group and non-urinary tract infection group).

A urinary tract infection (UTI) is diagnosed through clinical symptoms such as dysuria, frequency, urgency, suprapubic discomfort, or fever, confirmed by laboratory evidence. A positive urine culture indicating ≥100,000 colony-forming units (CFU)/ml of a single uropathogen in a clean-catch midstream sample indicated severe bacteriuria. Only symptomatic women exhibiting substantial bacteriuria were included in the analysis.

Finally, the researcher checked the participants' maternal and immediate newborn outcomes using paper medical records and electronic medical records.

During data collection, it was found that several participants' medical records lacked information on their monthly income and levels of education. After obtaining verbal agreement, the researcher contacted these participants and amended the records to incorporate the missing data. While data on all educational levels was acquired through this procedure, a significant portion of monthly income data was excluded due to participants being either unresponsive or inaccessible. Data about monthly income were missing for 516 participants from a total sample of 1,150. Therefore, the analysis was excluded for these missing values. Other sociodemographic variables (such as Age, place of residency, and occupational status) were available in the electronic medical records.

Two researchers conducted double data entry and pilot-tested the data abstraction form to ensure data completeness and accuracy. Original records were examined to address discrepancies, and 10% of the data underwent auditing by a secondary reviewer for validation.

### Instruments

A data abstraction form consisting of four sections was developed to collect data. The first section concerned socio-demographic data about the participants, including maternal Age, place of residency, educational level, occupational status, monthly income, obstetric history (such as fertility treatment and gestational Age at delivery), and medical and surgical history. The second section gathered information about immediate newborn health (such as gestational Age at the time of birth), Apgar score (at one, five, and ten minutes), gender of baby, growth parameters (including height, weight, head circumference, and chest circumference), admission to neonatal intensive care unit, reason for admission, and perinatal mortality. Section three relates to birth outcomes, such as the type of labour (spontaneous, induced, or augmented), the type of birth (vaginal, instrumental, or caesarean section), complications during labour (such as placental abruption, foetal distress, or failure to progress), and duration of hospitalisation after delivery. The form was subjected to content validity by three experts in obstetrics and epidemiology to guarantee clarity, comprehensiveness, and compatibility with the study objectives. The Data Collection Procedure section outlines that the form underwent pilot testing, duplicate data entry was conducted, and 10% of the data was independently audited by a secondary reviewer. These measures guaranteed the comprehensiveness, accuracy, and reliability of the extracted data.

### Data analysis procedure

The mean and standard deviation were employed to describe the metric continuous variables. The frequencies and percentage analysis were applied for categorically measured variables. The dependent variable was the occurrence of a urinary tract infection during pregnancy. The independent variables comprised education level, employment status, maternal Age, gestational Age at booking, and trimester of pregnancy. An interaction term between maternal age and educational level (university degree vs. high school or below) was created to examine whether the association between education and UTI occurrence varied by age. Multiple variables were identified as confounding factors in the multivariate model: percentage change in haemoglobin, maternal haemoglobin levels at booking and delivery, history of gestational hypertension, administration of blood transfusion for anaemia, and hospitalisation during pregnancy.

A multivariate logistic regression analysis was used to test the predictors of the women's odds of labour complications. The association between the predictor factors and the foetal and neonatal number of complications was expressed as an odds ratio with a 95% confidence interval. The SPSS IBM version 21 program was used for statistical data analysis, and the alpha significance level was considered at the 0.050 level.

## Results

### Socio-demographic characteristics of the study participants

The study encompassed 1,150 primigravida women with a singleton pregnancy. The mean age at booking was 26.4 ± 4.3 years, with 43.3% of the women aged between 21 and 25. A majority lived in Irbid (60.3%), had attained a minimum of a high school education (63%), and were employed (60.6%). The majority of women indicated that families earn between 500 and 1,000 JOD monthly. The proportion of individuals with a history of non-obstetric surgery was only 6.6%. The mean number of prenatal visits per pregnancy was seven, and the average gestational age at booking was 22.1 ± 7.5 weeks (Table 1).

**Table 1:** Descriptive analysis of the women's sociodemographic and obstetrical history, *n* = 1,150.

Variable	Frequency	Percentage
Mother's age (years)		26.42 (4.29)
Mothers age groups		
18–20 years	59	5.1
21–25 years	498	43.3
26–30 years	438	38.1
31–35 years	108	9.4
≥36 years	47	4.1
City of residence		
Irbid	693	60.3
Ramtha	210	18.3
Ajloun	94	8.2
Jarash	48	4.2
Mafraq	105	9.1
Educational level		
High school or below education	724	63
University degree	426	37
Employment		
Unemployed	453	39.4
Employed	697	60.6
Past surgical history		
No	1,074	93.4
Yes	76	6.6
Gestational age at hospital presenting time (weeks), mean (sd)		22.10 (7.48)
Number of antenatal visits		7.02 (2.53)
Serum hemoglobin level at first antenatal visit g/dl, mean (sd)		12.05 (0.71)
Serum hemoglobin level at baby delivery time g/dl, mean (sd)		11.67 (0.76)
Percentage gain in hemoglobin from baseline(booking time), mean (sd)		−2.89 (7.03)

### Prevalence of UTI among the study participants

The total prevalence of urinary tract infection (UTI) was 8.9% (*n* = 102), with annual rates of 8.5% in 2017, 6.5% in 2018, and 11.4% in 2019. The prevalence was greater among women with a high school level or lower (71.6%) compared to those with a university degree (28.4%). Women with urinary tract infections (UTIs) scheduled antenatal care sooner (18.9 ± 10.0 weeks) than those without UTIs (22.4 ± 7.1 weeks) and had lower mean hemoglobin levels at booking (11.73 ± 0.98 g/dl vs. 12.10 ± 0.67 g/dl) and at delivery (10.78 ± 1.25 g/dl vs. 11.76 ± 0.63 g/dl). Hospitalization during pregnancy, blood transfusions for anemia, and gestational hypertension were more prevalent among women with urinary tract infections. The prevalence of urinary tract infections (UTIs) during pregnant trimesters was 2.1% at booking, 2.3% in the first trimester, 2.8% in the second trimester, and 3.8% in the third trimester (Table 2).

**Table 2:** Bivariate analysis explaining the associated factors with the development of UTI.

Variable	UTI throughout pregnancy time		Test statistic		*p*-value
	No UTI (*n* = 1,048)	UTI (*n* = 102)			
Mother's age (years)	26.39 (4.23)	26.71 (4.92)	t(1,148) = 0.70		0.483
Mothers age groups					
18–20 years	53 (5.1)	6 (5.9)	*χ*2 (4) = 4.18	LR	0.382
21–25 years	455 (43.4)	43 (42.2)			
26–30 years	404 (38.5)	34 (33.3)			
31–35 years	97 (9.3)	11 (10.8)			
≥36 years	39 (3.7)	8 (7.8)			
City of residence					
Irbid	628 (59.9)	61 (59.8)	χ2 (5) = 4.99	LR	0.416
Alramtha	197 (18.8)	13 (12.7)			
Ajloun	85 (8.1)	9 (8.8)			
Jarash	41 (3.9)	7 (6.9)			
Mafraq	94 (9)	11 (10.8)			
Educational level					
High school level or lower education	651 (62.1)	73 (71.6)	χ2 (1) = 3.60		0.059
University degree	397 (37.9)	29 (28.4)			
Households monthly income					
<500 jod	171 (29.1)	14 (29.8)	χ2 (2) = 1.01	LR	0.605
500–1,000 jod	405 (69)	31 (66)			
>1,000 jod	11 (1.9)	2 (4.3)			
Employment					
Unemployed	420 (40.1)	33 (32.4)	χ2 (1) = 2.32		0.128
Employed	628 (59.9)	69 (67.6)			
Past surgical history					
No	980 (93.5)	94 (92.2)	χ2 (1) = 0.28		0.599
Yes	68 (6.5)	8 (7.8)			
Gestational age at hospital presenting time (weeks), mean (sd)	22.39 (7.12)	18.93 (9.97)	t(111.23) = 3.42		**0**.**001**
Number of antenatal visits	7.01 (2.49)	7.14 (2.87)	t(116.31) = 0.43		0.668
Serum hemoglobin level at first antenatal visit, mean (sd)	12.10 (0.67)	11.73 (0.98)	t(110.24) = 3.54		**0**.**001**
Serum hemoglobin level at baby delivery time, mean (sd)	11.76 (0.63)	10.78 (1.25)	t(106.03) = 7.52		**<0**.**001**
Percentage change in delivery time serum hemoglobin from baseline(booking time)	−2.42 (6.42)	−7.77 (10.39)	t(108.63) = 5.11		**<0**.**001**

Bold values indicate statistical significance at *p* < 0.05.

### Factors associated with the occurrence of UTI

In the multivariate logistic regression analysis (Table 3), increasing maternal age correlated with decreased odds of urinary tract infection (AOR = 0.904; 95% CI: 0.839–0.974; *p* = 0.008). Women having a university degree exhibited markedly reduced chances compared to those with a high school level or lower (AOR = 0.027; 95% CI: 0.002–0.344; *p* = 0.005). A significant interaction between age and education was noted (AOR = 1.139; 95% CI: 1.040–1.248; *p* = 0.005). Third-trimester pregnancy was linked to higher odds relative to the first trimester (AOR = 1.856; 95% CI: 1.052–3.273; *p* = 0.033). Additional significant predictors comprised blood transfusion for anemia (AOR = 3.662; 95% CI: 2.185–6.138; *p* < 0.001), hospitalization during gestation (AOR = 6.784; 95% CI: 4.075–11.292; *p* < 0.001), and gestational hypertension (AOR = 1.706; 95% CI: 1.047–2.779; *p* = 0.032). Every extra week of delay in antenatal booking decreased the likelihood of urinary tract infection (UTI) by 3.8% (AOR = .962; 95% CI: 0.935–0.990; *p* = 0.007), but each 1% rise in hemoglobin from booking to delivery diminished the odds by 5% (AOR = 0.950; 95% CI: 0.922–0.978; *p* = 0.001).

**Table 3:** Multivariate generalized estimating equation (GEE) model explaining mothers odds of having had urinary tract infection (UTI) throughout pregnancy period.

Parameter	Adjusted odds ratio (AOR)	95% CI for odds ratio		*p*-value
		Lower	Upper	
(Intercept)	.154	.019	1.268	0.082
Mother's Age (years)	.904	.839	.974	**0**.**008**
Mother's Educational Level = University Degree	.027	.002	.344	**0**.**005**
Interaction University Degree* Mother's Age	1.139	1.040	1.248	**0**.**005**
Employment = Yes	1.617	.934	2.799	0.086
Time = 3rd Trimester	1.856	1.052	3.273	**0**.**033**
Time = 2nd Trimester	1.270	.698	2.311	0.433
Received Blood for anemia during pregnancy = Yes	3.662	2.185	6.138	**<0**.**001**
Required hospitalization throughout pregnancy	6.784	4.075	11.292	**<0**.**001**
Having Had Gestational Hypertension	1.706	1.047	2.779	**0**.**032**
Maternal Gestational Age at booking time in weeks	.962	.935	.990	**0**.**007**
Mean gain (%) in serum HGB at delivery time from baseline (booking time)	.950	.922	.978	**0**.**001**
Dependent Variable: UTI at trimester (1–3).				

The interaction term between University Degree and Mother's Age is generated by multiplying these two variables to evaluate if the impact of education on UTI incidence differs according to maternal age.*.

Bold values indicate statistical significance at p < 0.05.

## Discussion

In the current study, the prevalence of urinary tract infections among primigravida women with singleton foetuses who attended King Abdullah University Hospital between 1st January 2017 and 1st January 2020 was 8.9%. Our found prevalence of 8.9% is between the greater global estimate of around 23.9% from recent systematic reviews and the lower regional value of roughly 5% reported in research from Riyadh, Saudi Arabia (2, 8). This diversity may signify differences in demographic traits, access to prenatal care, and screening techniques. The acknowledged benefits of asymptomatic bacteriuria (ASB) screening, emphasized in ACOG's 2023 clinical consensus, support the routine urine screening in early pregnancy to reduce complications like pyelonephritis and preterm birth (3).

This finding contrasts with previous studies conducted in the north of Jordan which reported that the prevalence of UTI among pregnant women was 20% and that UTIs were responsible for 10% of preterm births (20). The discrepancies can be attributed to the current study's population comprising primigravida women from a singular area, in contrast to the study population of Quteitat et al. (20) which included both multigravida and primigravida mothers from northern Jordan. Furthermore, in comparison to Quteitat et al. (20), the present study demonstrates a notable prevalence of maternal UTIs among primigravida women with singleton foetuses in one specific location.

The incidence of urinary tract infections in pregnant women globally varies. Research undertaken in Asian nations in 2011, specifically India and Turkey, indicated that the prevalence of maternal urinary tract infections was 25% and 9%, respectively (21). In 2013, Gilbert et al. reported a 40% prevalence of maternal UTIs in Nigeria, whereas in 2016, Shaheen et al. reported a 32% prevalence in Egypt. In 2008, the prevalence of urinary tract infections among pregnant women in the United States was 17% (21). The discrepancies among these studies may be attributed to changes in their demographic, context, and duration. The significant prevalence of urinary tract infections seen in this study may be related to insufficient knowledge regarding UTI prevention or noncompliance with existing measures.

The rise in UTI incidence noted in the second and third trimesters is attributed to many hormonal and physiological alterations occurring during pregnancy. The elevated levels of progesterone will induce relaxation of the smooth muscle in the urinary tract, which may lead to diminished bladder tone and urethral dilation. This may result in urine stasis, recognised as a risk factor for bacterial infection (22, 23). Moreover, pregnancy induces mechanical compression of the urinary system due to the enlarging foetus, which subsequently hinders urine flow and elevates the risk of infection (24). Furthermore, the immunological adjustments during pregnancy diminish cell-mediated immunity, resulting in reduced resistance to urinary infections; nevertheless, this adaptation is crucial for foetal tolerance (23).

Further, an association exists between low haemoglobin levels and urinary tract infections. Iron deficiency compromises adaptive immune function by diminishing T-cell activation, hence heightening susceptibility to infections, particularly UTIs (25). The current study has shown that hemoglobin levels were significantly reduced in pregnant women with urinary tract infections relative to those without such infections. Our results are consistent with previous research showing that iron deficiency can weaken the immune system and make infections more likely (25). This highlights the importance of checking hemoglobin levels during pregnancy and addressing anemia early to lower the risk of UTIs.

Our study found that multidrug-resistant bacteria, specifically ESBL-producing E. coli, were responsible for an increased incidence of UTIs in women, even in the absence of traditional risk factors. Comparable findings were noted in northern Jordan, where 62% of isolates from pregnant women were identified as ESBL-producing E. coli (26). This trend may signify the growing problem of community-acquired resistance in Jordan and highlights the need for improved antibiotic monitoring and comprehensive surveillance methods.

Furthermore, the findings of the current study indicated that advanced maternal age correlates with a diminished risk of urinary tract infections during gestation. Unlike the findings from Ethiopia and Saudi Arabia, where the prevalence of UTIs increased with age (*p* = 0.008 and *p* = 0.04, respectively) (27, 28). Also, our findings contrast with those of Amiri et al. (13), who reported no significant association between age and UTI prevalence (*p* > 0.05). Variations in study conditions, the classification of age groups, and changes in women's behaviours or cultural customs may clarify these inconsistencies.

Women with a university degree demonstrated a markedly lower probability of acquiring a urinary tract infection during pregnancy (OR = 0.027; 95% CI: 0.002–0.344; *p* = 0.005) in comparison to those with a high school education or less. This corresponds with the results of Derese et al. (27), which linked reduced literacy to increased UTI incidence (*p* = 0.02), and with a systematic review and meta-analysis from Iran that exhibited a same pattern (29). In contrast, Elkashif (28) found no significant link between education and the prevalence of UTIs (*p* = 0.28). A possible explanation is that women with higher educational levels may uphold better hygiene and exhibit greater diligence over nutrition and supplement consumption throughout pregnancy.

This study found no significant link between residential location and the incidence of urinary tract infections. All participants in this study reside in urban regions and have access to diverse healthcare alternatives for pregnant women. Derese et al. (27) noted a higher incidence of urinary tract infections in urban areas compared to rural regions; in contrast, Shaheen et al. (30) indicated a greater prevalence of urinary tract infections in rural settings (*p* = 0.000).

This study did not identify any significant correlation between occupational status, monthly household income, and the incidence of UTI during pregnancy (*p* > 0.05). The findings of this study do not align with those of previous studies. For example, Derese et al. (27) found that pregnant women with a monthly income between 501 and 1000 birr (50%) exhibited the highest incident rate of UTIs followed by those with an income of less than 500 birr (34.6%). Additionally, the same study identified a significant difference between the urinary tract infection and non-urinary tract infection groups (*p* = 0.009 and *p* = 0.025, respectively). Furthermore, Elkashif (28) found that 57.4% of women in the urinary tract infection group were workers (compared to 29.8% of women in the non-urinary tract infection group), and this difference was statistically significant (*p* = 0.005). The differences between the findings of this study and the aforementioned research may be caused by variations in daily working hours, economic conditions of the countries, and the health insurance coverage available to pregnant women throughout the study environments.

### Implications

The findings of this study indicate the need to introduce a range of measures to prevent maternal urinary tract infections. For example, pregnant women should be assessed for particular risk factors highlighted in this study during antenatal care, including delayed antenatal booking, pregnancy-related complications requiring hospitalization, and hemoglobin levels. Healthcare professionals should develop health education programs that enable women to identify early symptoms and receive dietary counseling to prevent anemia. Moreover, the importance of personal cleanliness habits must be underscored, along with the necessity of regular prenatal examinations and urine screenings. These interventions can be incorporated into standard antenatal care and administered by nurses and midwives to enhance maternal health and decrease the prevalence of UTIs during pregnancy.

### Recommendations

Early detection and treatment of UTIs can avert the onset of pyelonephritis (31); therefore, this study recommends first-trimester screening for asymptomatic urinary tract infections for all pregnant women. Furthermore, every pregnant woman must be evaluated for health risk issues during antenatal care and asked about their health risk factors. Additionally, healthcare providers should advise pregnant women to attend follow-up appointments and undergo urine analysis and culture during each trimester (as well as when necessary) to facilitate the timely identification of urinary tract infections. Urinary tract infections are a global concern; therefore, further research should build on the current study by employing different research designs, analysing larger study populations, and studying different geographical regions across Jordan as well as private and government-run hospitals.

### Limitations

This study contains two major limitations. First, the study's retrospective design may compromise the completeness and accuracy of the data. Information bias may arise from incomplete records—specifically, unrecorded UTIs or related risk factors—which could result in underreporting, hence introducing selection bias and potentially compromising the generalizability of the study findings.

Second, this approach limits the ability to determine causality and may be vulnerable to absent or inadequate data (19)

## Conclusion

This study found the incidence of urinary tract infection was 8.9% and the incidence of recurrent UTI was 3.7% (from the total sample) and 42.2% (from the UTI group). Maternal Age and educational level are risk factors for UTI among primigravida women.

Based on these results, the incidence rate of UTI is significant and highlights the need for the introduction of effective education programmes for pregnant women regarding their personal hygiene, nutritional habits, sexual practices, adherence to screening tests, and urinary tract infection-related complications. The introduction of such measures may help to reduce the incidence of urinary tract infections during pregnancy.

## Data Availability

The raw data supporting the conclusions of this article will be made available by the authors, without undue reservation.

## References

[1] American Urological Association. Adult UTI (2019). Available online at: https://www.auanet.org/education/auauniversity/for-medical-students/medical-students-curriculum/medical-1student-curriculum/adult-uti (Accessed August 1, 2025).

[2] Salari N Khoshbakht Y Hemmati M Khodayari Y Khaleghi AA Jafari F Global prevalence of urinary tract infection in pregnant mothers: a systematic review and meta-analysis. Public Health. (2023) 224:58–65. 10.1016/j.puhe.2023.08.016 37734277

[3] American College of Obstetricians and Gynecologists. Urinary Tract Infections in Pregnant Individuals. Washington, DC: American College of Obstetricians and Gynecologists (2023). Available online at: https://www.acog.org/clinical/clinical-guidance/clinical-consensus/articles/2023/08/urinary-tract-infections-in-pregnant-individuals (Accessed August 8, 2023).

[4] Franklin TL Monif GR. Trichomonas vaginalis and bacterial vaginosis. Coexistence in vaginal wet mount preparations from pregnant women. J Reprod Med. (2000) 45(2):131–4. 10710744

[5] Mittal P Wing DA. Urinary tract infections in pregnancy. Clin Perinatol. (2005) 32(3):749–64. 10.1016/j.clp.2005.05.006 16085031

[6] Mavi A Rathi I Shannawaz M Saeed S Hasan S. Correlates of urinary tract infections among women of reproductive age in India: a systematic review. Cureus. (2024) 16(4):e58681. 10.7759/cureus.58681 38774177 PMC11107389

[7] de Souza HD Diório GRM Peres SV Francisco RPV Galletta MAK. Bacterial profile and prevalence of urinary tract infections in pregnant women in Latin America: a systematic review and meta-analysis. BMC Pregnancy Childbirth. (2023) 23(1):774. 10.1186/s12884-023-06060-z 37940852 PMC10631168

[8] Barnawi Y Alghamdi A Ibrahim A Al-Anazi L Alhumaida G Alotaibi R Prevalence of urinary tract infections in pregnant women and antimicrobial resistance patterns in women in Riyadh, Saudi Arabia: a retrospective study. BMC Infect Dis. (2024) 24(1):502. 10.1186/s12879-024-09385-y 38762526 PMC11102606

[9] Al-Issa M. Urinary tract infection among pregnant women in north Jordan Jordan university of science and technology (Master’s thesis). (2008).

[10] Emiru T Beyene G Tsegaye W Melaku S. Associated risk factors of urinary tract infection among pregnant women at felege hiwot referral hospital, bahir dar, north west Ethiopia. BMC Res Notes. (2013) 6:292. 10.1186/1756-0500-6-292 23885968 PMC3750516

[11] Matuszkiewicz-Rowińska J Małyszko J Wieliczko M. Urinary tract infections in pregnancy: old and new unresolved diagnostic and therapeutic problems. Arch Med Sci. (2015) 11(1):67–77. 10.5114/aoms.2013.39202 25861291 PMC4379362

[12] Osmundson SS Nickel KB Shortreed SM Dublin S Stwalley D Durkin MJ First-Trimester antibiotic use for urinary tract infection and risk of congenital malformations. JAMA Network Open. (2025) 8(7):e2519544–e2519544. 10.1001/jamanetworkopen.2025.19544 40632535 PMC12242685

[13] Amiri M Lavasani Z Norouzirad R Najibpour R Mohamadpour M Nikpoor AR Prevalence of urinary tract infection among pregnant women and its complications in their newborns during the birth in the hospitals of Dezful City, Iran, 2012–2013. Iran Red Crescent Med J. (2015) 17(8):e26946. 10.5812/ircmj.26946 26430526 PMC4585427

[14] Biggs WS Williams RM. Common gynecologic infections. Prim Care. (2009) 36(1):33–51. viii. 10.1016/j.pop.2008.10.002 19231601

[15] Genc MR Ford CE. The clinical use of inflammatory markers during pregnancy. Curr Opin Obstet Gynecol. (2010) 22(2):116–21. 10.1097/GCO.0b013e3283374ac8 20139764

[16] Sujith S Solomon AP Rayappan JBB. Comprehensive insights into UTIs: from pathophysiology to precision diagnosis and management. Front Cell Infect Microbiol. (2024) 14:1402941. 10.3389/fcimb.2024.1402941 39380727 PMC11458535

[17] Ministry of Health. (2019). The National Maternal Mortality Report 2018: Jordan, Towards Eliminating Preventable Maternal Deaths*.* Amman, Jordan. M. o. H. (Jordan).

[18] Data Commons. Jordan: Country in Asia, World. (2025) Available online at: https://datacommons.org/place/country/JOR?utm_medium=explore&mprop=count&popt=Person&hl=en (Accessed July 20, 2025).

[19] Polit DF Beck CT. Nursing Research: Generating and Assessing Evidence for Nursing Practice. 11th ed. Philadelphia, PA: Wolters Kluwer (2021).

[20] Quteitat A Shraideh I Malek AMA Gowieri A Alnashash H Amarin ZO. Maternal morbidity: results of a country-wide review. Arch Gynecol Obstet. (2012) 286(6):1357–62. 10.1007/s00404-012-2458-4 22805977

[21] Gilbert NM O'Brien VP Hultgren S Macones G Lewis WG Lewis AL. Urinary tract infection as a preventable cause of pregnancy complications: opportunities, challenges, and a global call to action. Glob Adv Health Med. (2013) 2(5):59–69. 10.7453/gahmj.2013.061 24416696 PMC3833562

[22] Committee on Clinical Consensus—Obstetrics, Graseck A Thompson JL Bryant AS Cahill AG Silverman NS Urinary tract infections in pregnant individuals. Obstet Gynecol. (2023) 142(2):435–45. 10.1097/aog.0000000000005269 37473414

[23] Kałuziak P Parys J Mikosińska A Kaźmierczak M Jajczak M Mossakowski M Review of urinary tract infections in pregnancy: risks, complications and management. Quality in Sport. (2025) 37:57277. 10.12775/QS.2025.37.57277

[24] Hatamleh R Al-Trad A Abuhammad S Aljabari M Joseph R. Urinary tract infection among pregnant Jordanian women: role of hygiene and sexual practices. BMC Pregnancy Childbirth. (2024) 24(1):694. 10.1186/s12884-024-06902-4 39443883 PMC11515754

[25] Stoffel NU Drakesmith H. Effects of iron Status on adaptive immunity and vaccine efficacy: a review. Adv Nutr. (2024) 15(6):100238. 10.1016/j.advnut.2024.100238 38729263 PMC11251406

[26] Al Momani W Elayan A Al Titi R Malkawi I Al momani L Al-Magableh M. Extended spectrum *β*-lactamase (ES*β*L)-producing E. coli causing urinary tract infection among pregnant women and pediatric patients in public hospitals in northern Jordan. PLoS One. (2025) 20(3):e0320292. 10.1371/journal.pone.0320292 40163505 PMC11957286

[27] Derese B Kedir H Teklemariam Z Weldegebreal F Balakrishnan S. Bacterial profile of urinary tract infection and antimicrobial susceptibility pattern among pregnant women attending at antenatal clinic in Dil Chora Referral Hospital, Dire Dawa, Eastern Ethiopia. Ther Clin Risk Manag. (2016) 12:251–60. 10.2147/tcrm.s99831 26937197 PMC4762443

[28] Elkashif M. Urinary tract infection among pregnant women and its associated risk factors: a cross-sectional study. Biomed Pharmacol J. (2019) 12:2003–10. 10.13005/bpj/1832

[29] Azami M Jaafari Z Masoumi M Shohani M Badfar G Mahmudi L The etiology and prevalence of urinary tract infection and asymptomatic bacteriuria in pregnant women in Iran: a systematic review and meta-analysis. BMC Urol. (2019) 19(1):43. 10.1186/s12894-019-0454-8 31146773 PMC6543660

[30] Shaheen HM El-Hakeem Hammad NA Farahat TM. Prevalence of urinary tract infection among pregnant women and possible risk factors. Menoufia Med J. (2016) 29(4):1054–9. 10.4103/1110-2098.202505

[31] World Health Organization. WHO recommendation on antibiotics for asymptomatic bacteriuria (2016). Available online at: https://www.who.int/publications/i/item/WHO-RHR-16.24 (Accessed June 8, 2025).

